# A retrospective study of factors associated with the development of oral candidiasis in patients receiving radiotherapy for head and neck cancer: Is topical steroid therapy a risk factor for oral candidiasis?

**DOI:** 10.1097/MD.0000000000013073

**Published:** 2018-11-02

**Authors:** Yumiko Kawashita, Madoka Funahara, Masako Yoshimatsu, Noriko Nakao, Sakiko Soutome, Toshiyuki Saito, Masahiro Umeda

**Affiliations:** a Oral Management Center, Nagasaki University Hospital; b Kyushu Dental University School of Oral Health Sciences, Fukuoka; c Department of Oral Health; d Department of Clinical Oral Oncology, Nagasaki University Graduate School of Biomedical Sciences, Nagasaki, Japan.

**Keywords:** head and neck cancer, oral candidiasis, radiotherapy, retrospective study, supportive care

## Abstract

The aims of this study were to investigate the incidence and risk factors for oral candidiasis in patients receiving radiotherapy for head and neck cancer, and to determine the influence of topical steroid therapy on the development of oral candidiasis.

We conducted a retrospective study of 300 patients receiving radiotherapy to the head and neck region. The primary endpoint was the incidence of oral candidiasis during radiotherapy. Associations between the incidence of oral candidiasis and various clinical factors were investigated. The cumulative incidence rate of oral candidiasis was calculated using the Kaplan–Meier method and analyzed by the log-rank test and Cox regression. Propensity score-matched analysis was used to assess the influence of topical steroid therapy on the development of oral candidiasis.

Oral candidiasis occurred in 75 (25.0%) of the 300 patients. Multivariate analysis identified minimum lymphocyte count and severity of oral mucositis during radiotherapy as independent risk factors for the development of oral candidiasis. Topical steroid therapy for oral mucositis was not associated with the incidence of oral candidiasis according to multivariate and propensity score matching analyses.

Oral candidiasis was associated with the suppression of the host's immunity and severe oral mucositis, but not topical steroid therapy. Proper oral health care during radiotherapy and the prevention of severe oral mucositis may reduce the incidence of oral candidiasis.

## 1 Introduction

Oral candidiasis is an opportunistic infection affecting oncologic patients.^[1]^ Head and neck cancer patients who receive radiotherapy with or without chemotherapy, in particular, are more susceptible to *Candida* colonization and infection.^[2]^ Under normal conditions, fungal organisms coexist with the other microorganisms of the normal oral flora and do not cause disease.^[3]^ However, changes in the oral and/or systemic environment can result in an overgrowth of fungal species, leading to clinical oral fungal infections. These changes include immunosuppression, an imbalance in the oral flora, hyposalivation, and local tissue damage. Finally, the incidence of oral candidiasis increases with aggressive systemic treatments^[4]^ and has been reported to affect approximately 40% of patients treated with chemotherapy and up to 100% of patients receiving cervical radiotherapy.^[5]^

The incidence and severity of oral mucositis also increase with aggressive systemic treatments including taxanes and antipyridermal growth factor receptor (EGFR).^[4]^ Oral mucositis provides favorable conditions for the development of oral candidiasis. *Candida*, commensal yeast of the digestive tract, is capable of colonizing mucositis lesions and infecting the oral mucosa.^[6]^ Overall, 60% to 90% of mucositis lesions are infected by *Candida*.^[7]^

The National Comprehensive Cancer Network Clinical Practice Guidelines for Head and Neck Cancer (Version 2.2017)^[8]^ provide that the goals of dental management during radiotherapy are to evaluate oral candidiasis and treat as clinically indicated. However, concrete recommendations for oral management methods are not informed and none of the methods were considered to be effective for the prevention of oral candidiasis.

In Nagasaki University Hospital, patients received radiotherapy to the head and neck region with oral management, including instructions regarding proper oral health care, spacer covering the entire dentition to prevent scatter radiation, professional tooth cleaning, oral rinsing or the use of commercial saliva substitutes, and topical steroid therapy for oral mucositis.^[9]^ However, there are some opinions that topical administration of steroids for oral mucositis should be avoided owing to concerns that oral candidiasis may occur.^[10]^ There is no established method for managing radiotherapy-induced oral mucositis.^[11–14]^

The aims of this study were to investigate the incidence and risk factors for oral candidiasis in patients receiving radiotherapy for head and neck cancer, and to determine the influence of topical steroid therapy on the development of oral candidiasis.

## 2 Methods

### 2.1 Setting and design

We conducted a retrospective study of 307 patients who received oral management associated with radiotherapy to the head and neck region, with or without chemotherapy, in the Perioperative Oral Management Center at Nagasaki University Hospital (Nagasaki, Japan) between July 2011 and May 2017. Medical records were reviewed to screen the study subjects. Among them, seven patients were excluded: 2 patients had taken antifungal agents during the 2 weeks prior to and/or during radiotherapy for prevention purposes, 2 patients did not have the dose-distribution chart for radiotherapy, 2 patients did not have blood tests, and one patient did not receive oral management in the middle of the radiotherapy. The remaining 300 patients included those who received radiotherapy to part of or the entire oral cavity, those who received oral management during radiotherapy, those who had complete medical records. The study was approved by the Institutional Review Board (approval number: 17071013) of Nagasaki University Hospital (Nagasaki, Japan). The need for informed consent was waived due to the retrospective nature of the study.

### 2.2 Oral management associated with radiotherapy

All patients were evaluated prior to oncological treatment as part of routine clinical practice by means of an oral and dental screening, including a radiographic examination. To prevent osteoradionecrosis, any infected teeth were extracted before radiotherapy.^[15]^ During radiotherapy, the patients’ teeth were covered by spacers to prevent scatter radiation, especially from any metallic restorations and enamel surface exposed to the oral mucosa, except in edentulous jaws.^[9]^ Patients received professional oral care by a dental hygienist at least once a week until the end of radiotherapy. The oral care method included the removal of dental plaque via professional mechanical tooth cleaning methods and gentle removal of mucosal debris with a water drenched sponge, to keep the oral cavity as clean as possible. Oral rinsing with 4% sodium gualenate hydrate solution and oral moisturizing gels were performed at least four times daily. When symptoms of radiotherapy-induced oral mucositis occurred (erythema, patchy ulcerations, or pseudomembranes of the mucosa), 1.0 mg/g dexamethasone ointment softened with olive oil was applied to the oral mucosa four times daily, after meals and before bedtime. Topical steroid ointment was not administered to patients with tumors of the oral cavity or oropharyngeal mucosa, because topical steroid therapy may attenuate the effect of radiotherapy on the tumor.

### 2.3 Outcome

Oral candidiasis was confirmed by therapeutic diagnosis. In other words, the clinical feature of oral candidiasis was characterized by the presence of white papular lesions that were removed by a piece of gauze, revealing an underlying erythema. Patients were immediately followed by topical antifungal treatment and symptomatic improvement of the oral candidiasis was observed. The diagnosis of oral candidiasis was not necessarily based on bacterial tests.

### 2.4 Data collection

Demographic and clinical characteristics were collected from medical records. The variables examined included: Demographic factors (age, sex, diabetes mellitus), tumor-related factors (primary site, stage, and pathology), treatment-related factors (radiotherapy method, radiotherapy dose, concomitant chemotherapy, irradiated area of the oral cavity and parotid gland), laboratory test results (minimum neutrophil and lymphocyte counts during radiotherapy), the use of dexamethasone ointment softened with olive oil, and oral mucositis.

The radiotherapy method was divided into conventional three-dimensional conformal radiotherapy and intensity-modulated radiotherapy. Concomitant chemotherapy was classified into three categories: none (radiotherapy alone), cisplatin or carboplatin (CRT), and cetuximab (BRT). Irradiated areas of the oral cavity (less than two-thirds or two-thirds or more) and the parotid gland (unilateral or bilateral) were identified using dose-distribution charts. Oral mucositis was graded according to the National Cancer Institute Common Terminology Criteria for Adverse Events (Version 3.0).^[16]^ The criteria for oral mucositis were as follows: Grade 1: erythema of the mucosa; Grade 2: patchy ulcerations or pseudomembranes; Grade 3: confluent ulcerations or pseudomembranes, bleeding with minor trauma; Grade 4: tissue necrosis, significant spontaneous bleeding, life-threatening consequences; Grade 5: death. Minimum neutrophil and lymphocyte counts were recorded during radiotherapy. Neutrophil and lymphocyte counts were categorized into 2 groups: Grade ≤2 or Grade ≥3, according to the National Cancer Institute Common Terminology Criteria for Adverse Events (Version 4.0).^[17]^ The criteria for neutrophil counts included: Grade 1, >1500 /mm^3^; Grade 2, 1000–1500 /mm^3^; Grade 3, 500–1000 /mm^3^; and Grade 4, <500 /mm^3^. The criteria for lymphocyte counts included: Grade 1, >800 /mm^3^; Grade 2, 500–800 /mm^3^; Grade 3, 200–500 /mm^3^; and Grade 4, <200 /mm^3^.

### 2.5 Statistical analyses

Quantitative variables are presented as the number, mean, standard deviation, and range. Qualitative variables are presented as the number and percentage. The incidence of oral candidiasis was compared between subgroups using the Chi-square test, Student's *t*-test, and Welch test. The cumulative incidence rate of oral candidiasis was calculated using the Kaplan–Meier method and analyzed by the log-rank test and univariate Cox regression analysis. A multivariate Cox proportional hazards model was used to evaluate all predictive factors with a forward stepwise approach.

Next, a propensity score-matched analysis was used to reduce the effects of confounders and to evaluate the effect of topical steroid therapy on the development of oral candidiasis. Propensity scores were calculated for all patients by logistic regression analysis of all 15 variables associated with the treatment method. The concordance index (c-index) was 0.883, which indicated a strong ability to differentiate between patients with topical steroid therapy or without, and the Hosmer–Lemeshow statistic was insignificant (*P = *.328), indicating good calibration. The propensity scores, which reflected the probability that a patient would receive topical steroid therapy, ranged from 0.04704 to 0.94876 in the group with topical steroid therapy and from 0.02975 to 0.91078 in the group without topical steroid therapy. The 82 propensity score-matched cases (those who received topical steroid therapy versus those who did not) were then evaluated by univariate analysis to identify the factors associated with the development of oral candidiasis outcome.

Statistical analyses were conducted using Statistical Package for the Social Sciences for Windows (Version 24; IBM Corp., Tokyo, Japan). A 2-sided *P* < .05 considered as statistically significant.

## 3 Results

The study participants consisted of 300 patients, the clinical characteristics of whom are presented in Table 1. Of the 300 patients, 225 were male and 75 were female. The age of the patients ranged from 31 to 92 years, with a mean age of 67 years. The primary tumor site was the head and neck in 275 patients (91.7%). More than 80% of the patients had an advanced tumor stage. BRT/CRT was observed in 199 patients (66.3%). All patients received radiotherapy to part of or the entire oral cavity. With respect to the radiotherapy method, 252 patients (84.0%) underwent three-dimensional conformal radiotherapy, whereas 48 patients (16.0%) underwent intensity modulated radiotherapy. The mean total dose of radiotherapy was 60.0 Gy. Radiotherapy was completed in 272 patients (90.7%). Grade 2/3 oral mucositis occurred in 209 patients (69.7%). None of the patients had Grade 4/5 oral mucositis. Topical steroid therapy was administered to 176 patients (58.7%). Oral candidiasis occurred in 75 (25.0%) of the 300 patients. The univariate analysis revealed that total radiotherapy dose, radiotherapy method, oral mucositis, topical steroid therapy, and minimum lymphocyte count during radiotherapy were significantly associated with the incidence of oral candidiasis.

**Table 1:**
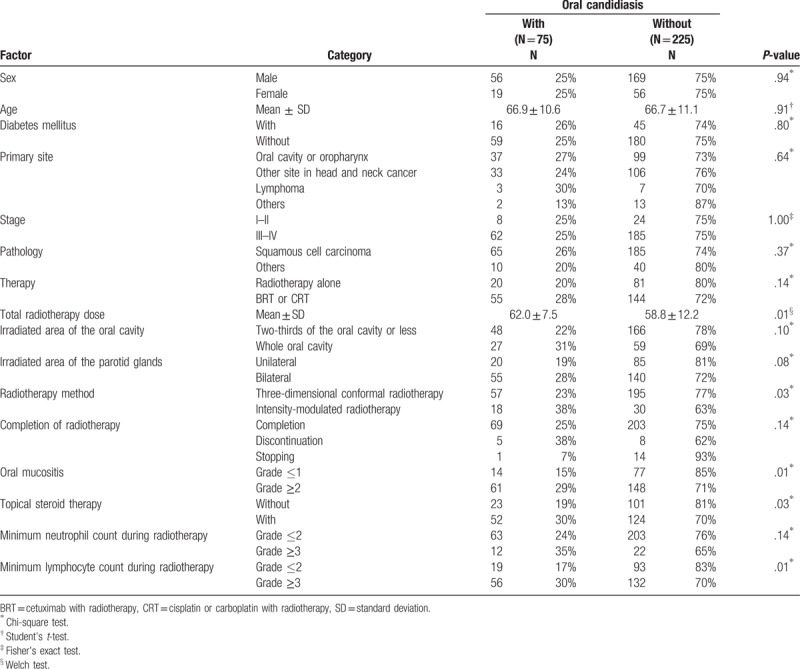
Characteristics of patients receiving radiotherapy with incidence of oral candidiasis.

**Figure 1:**
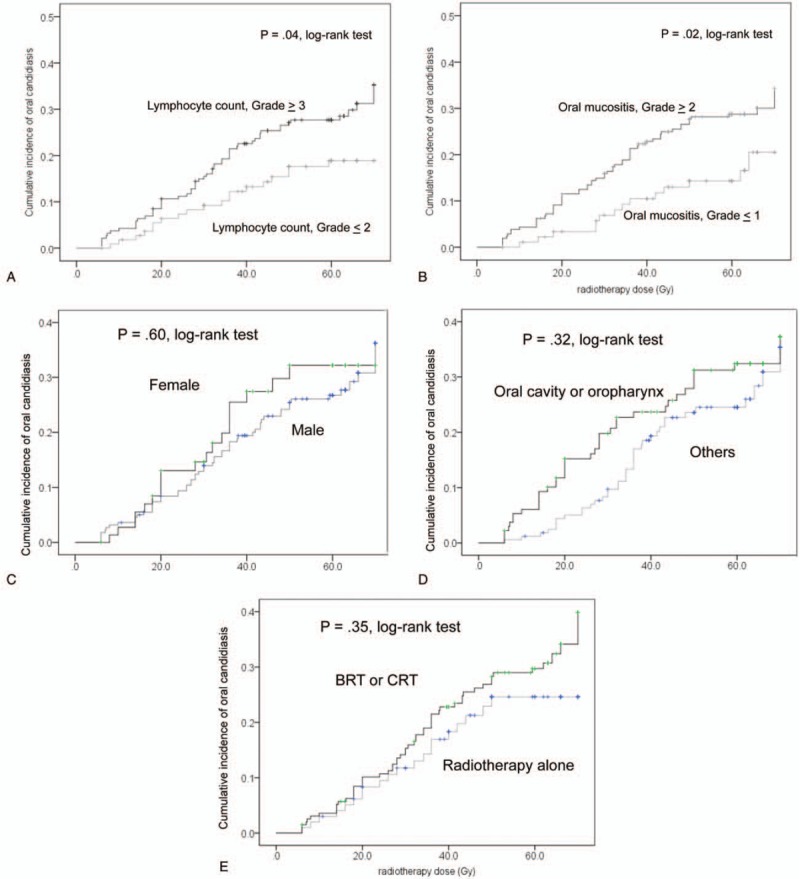
Kaplan–Meier curves of the cumulative incidence rates of oral candidiasis. (A) Minimum lymphocyte count during radiotherapy; (B) grade of oral mucositis; (C) sex; (D) primary site; (E) therapy. BRT = cetuximab with radiotherapy, CRT = cisplatin or carboplatin with radiotherapy.

**Figure 2:**
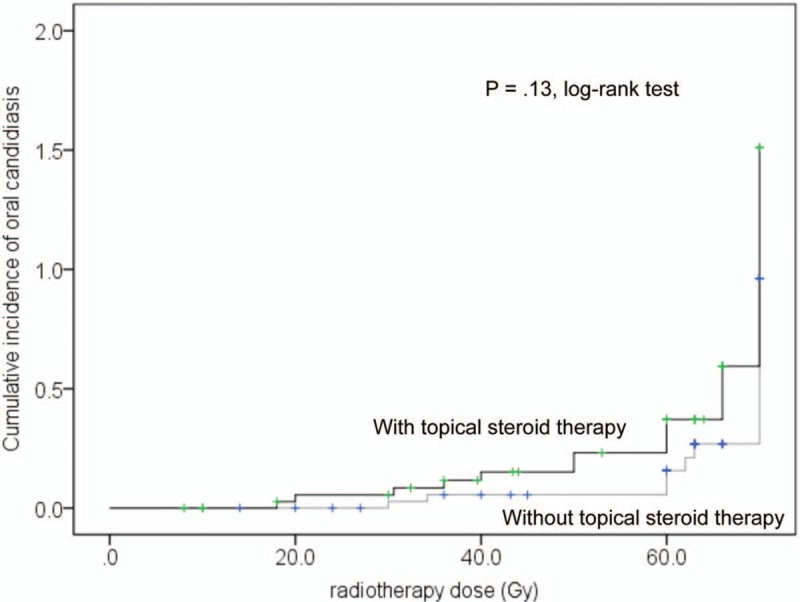
Kaplan–Meier curves of the cumulative incidence rates of oral candidiasis according to topical steroid therapy in 82 propensity score-matched patients.

Univariate Cox regression analysis revealed that the minimum lymphocyte count and severity of oral mucositis during radiotherapy were significantly associated with the incidence of oral candidiasis (Table 2). Oral candidiasis was more frequent in patients with irradiation of the entire oral cavity, or those who were treated with topical steroids. However, it was not significantly different from that of patients without oral candidiasis. Multivariate analysis identified minimum lymphocyte count (Grade 3) (hazard ratio: 1.72, 95.0% confidence interval: 1.01–2.95) and Grade 2/3 oral mucositis during radiotherapy (hazard ratio: 1.92, 95.0% confidence interval: 1.07–3.44) as independent risk factors for the development of oral candidiasis (Table 2). The cumulative incidence rates of oral candidiasis according to the minimum lymphocyte count and oral mucositis are shown in Figure 1A and B, respectively. Each Figure 1C to E shows that the cumulative incidence rates of oral candidiasis according to sex, primary site, and therapy which were not significantly associated with the incidence of oral candidiasis. Topical steroid therapy was not significantly associated with the incidence of oral candidiasis. Furthermore, propensity score-matched analysis was performed, and we found that variables were not significantly associated with the use of topical steroids in 82 patients (Table 3). Consequently, the incidence of oral candidiasis was not associated with topical steroid therapy (Table 4). According to the Kaplan–Meier method, the incidence rate of oral candidiasis was not associated with topical steroid therapy (*P* = .13; Fig. 2).

**Table 2:**
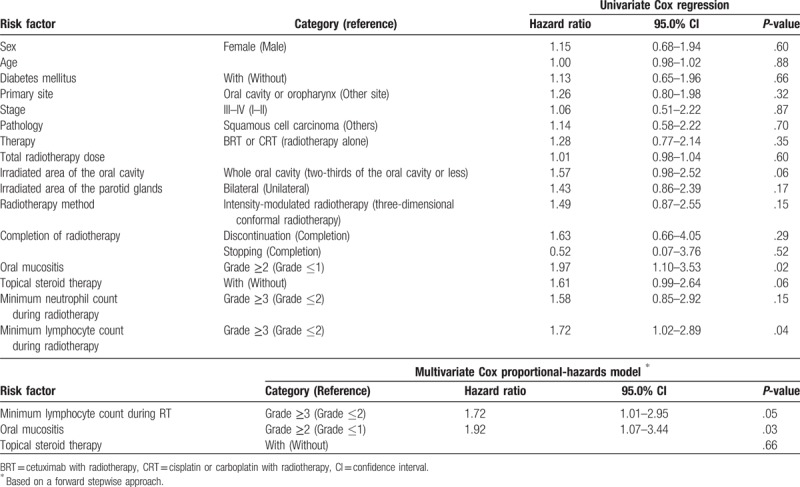
Univariate and multivariate analysis of risk factors for oral candidiasis.

**Table 3:**
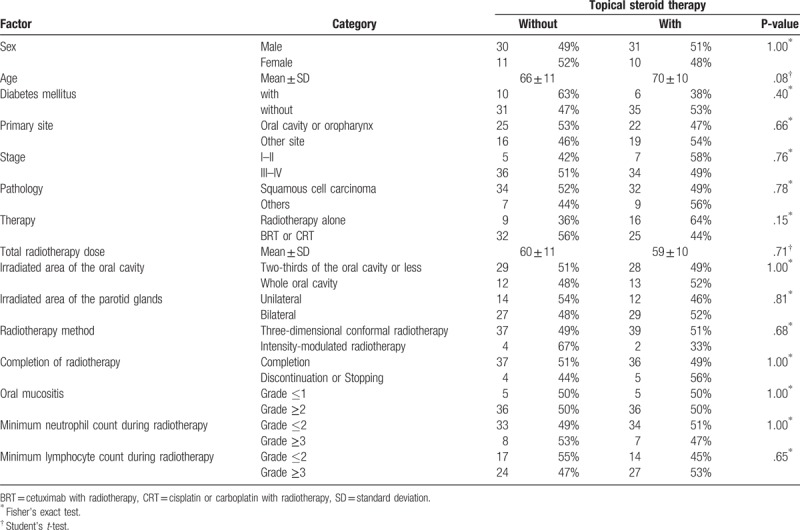
Characteristics of with or without topical steroid therapy groups comprising 82 propensity score-matched patients.

**Table 4:**
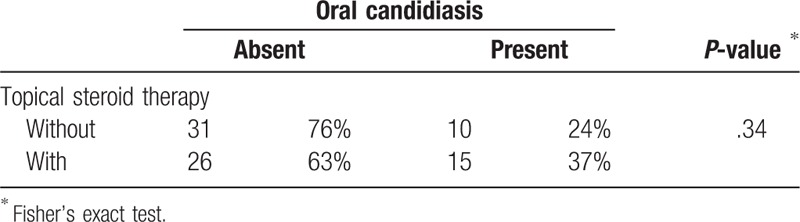
Univariate analysis of the association between the incidence of oral candidiasis and topical steroid therapy for oral mucositis in 82 propensity score-matched patients.

## 4 Discussion

In this retrospective study of 300 patients who received radiotherapy for head and neck cancer with oral management, we revealed that the incidence rate of oral candidiasis during radiotherapy was 25.0%, and that this was significantly associated with the minimum lymphocyte count and severity of oral mucositis. Moreover, topical steroid therapy for oral mucositis was not associated with oral candidiasis.

A systematic review^[3]^ reported that the weighted prevalence of clinical oral fungal infections in observational studies was 37.4% of patients receiving head and neck radiotherapy alone and 66.7% of patients receiving head and neck radiotherapy with chemotherapy. However, these studies reported data on only 10 to 63 patients. A large-scale study clarified that the prevalence of oropharyngeal candidiasis was 30.1% of 123 head and neck cancer patients among 2,042 patients with solid tumors and/or lymphomas treated with cancer therapy.^[1]^ However, this prior study did not demonstrate oral care management during radiotherapy. No study has investigated the incidence of oral candidiasis during radiotherapy to the head and neck region with oral management.

Oral candidiasis occurs as a result of an imbalance between fungal virulence factors and host defenses. A review^[2]^ showed that risk factors for oropharyngeal candidiasis in head and neck cancer patients were oral tumor, comorbidities (tobacco use, poor oral hygiene, and oral prostheses), xerostomic, antibacteria, immunosuppression, myelosuppression, and hyposalivation. An increased risk of clinical disease is observed in individuals who smoke tobacco products, and in patients using local or systemic steroid medications and antibiotics.^[2]^ However, the underlying mechanisms are not well understood. Our study showed that the incidence of oral candidiasis was associated with a low lymphocyte count during radiotherapy, and it is suggested that oral candidiasis is associated with the suppression of the host's immunity. Smoking has been shown to be a risk factor for oral candidiasis. However, we did not evaluate smoking as a risk factor. The reason for this is that, since smoking is a risk factor for the development of head and neck cancer,^[18]^ current smoking patients receive antismoking education before radiotherapy at Nagasaki University Hospital. Therefore, 199 (66.3%) participants in this study received BRT/CRT during hospitalization, and thus, could not smoke. Furthermore, a prior study^[19]^ identified that smoking was not a statistically significant risk factor for increased candidal colonization in patients undergoing oral and pharyngeal radiation therapy. In addition, we did not evaluate antibiotics as a risk factor for the incidence of oral candidiasis, because almost all patients did not receive systemic administration of antibiotics.

In addition to the above-mentioned risk factors, a significant correlation between the reduction of saliva flow rates, candidal colonization, and clinical presentation was detected in patients who received radiotherapy for head and neck cancer.^[20]^ Salivary antimicrobial factors include lysozymes, lactoferrins, histatins, defensins, and antibodies (secretory immunoglobulin A), which inhibit fungal colonization,^[21]^ including epithelial adhesion.^[22]^ Any qualitative or quantitative change in saliva may impact resident bacterial flora and promote the proliferation of yeast.^[2]^ We did not evaluate the salivary flow rate. However, we did evaluate the irradiated area of the parotid gland. We hypothesized that the irradiated area of the bilateral parotid glands resulted in a severely dry mouth compared to the irradiated area of the unilateral parotid gland. However, the irradiated area of the parotid gland was not significantly associated with the incidence of oral candidiasis.

Furthermore, we evaluated neutrophil and lymphocyte counts during radiotherapy as a risk factor of myelosuppression; and grading of oral mucositis and lymphocyte count during radiotherapy as a risk factor of immunosuppression. All patients received professional oral care at least once a week to keep the oral cavity as clean as possible from the onset of radiotherapy. Moreover, we educated patients to remove the oral prostheses at bedtime or when they were damaged by radiotherapy induced oral mucositis; and to keep their oral prostheses in a drug solution. Therefore, we did not evaluate for poor oral hygiene and oral prostheses.

Interestingly, our results showed that the incidence of oral candidiasis was associated with Grade 2/3 oral mucositis. It is likely that severe oral mucositis cannot provide a barrier to infection for oral candidiasis. The incidence rate of oral mucositis was high in head and neck cancer patients treated with radiotherapy, ranging from 50% to 90%, depending on the radiotherapy field, dose, fractionation, and administration of chemotherapy.^[23]^ Nevertheless, a consensus protocol for prophylaxis or the treatment of oral mucositis has not yet been established.^[11–14]^ We treat radiation-induced oral mucositis using topical steroids because it has an anti-inflammatory effect on oral mucositis. Rugo et al^[24]^ showed that the prophylactic use of dexamethasone mouthwash substantially reduced the incidence and severity of everolimus-related stomatitis in patients with breast cancer. Moreover, we use dexamethasone ointment softened with olive oil to settle extensive radiation-induced oral mucositis as topical steroid therapy. In addition to topical steroid therapy for chemotherapy-induced oral mucositis, a review of conventional and novel therapy for the management of oral lichen planus^[25]^ concluded that no treatment is superior to topical steroids as first-line therapy. If we could find out how to prevent severe oral mucositis with topical steroid therapy, the incidence of oral candidiasis may reduce.

A review of topical corticoids in oral pathology^[26]^ concluded that oral candidiasis occurred in 25–55% of cases, and that local adverse effects and the frequency of presenting candidiasis was directly related to the use of stronger topical corticoids in aqueous forms, over prolonged periods of time, and at high concentrations. However, this review did not provide an experimental evidence. Al-Hashimi et al^[27]^ concluded that not all studies with corticosteroids reported side effects. When reported, oral candidiasis was the most frequent side effect. Side effects were more prevalent with systemic corticosteroids than topical agents. The overall conclusions suggest that corticosteroids are effective in the management of oral lichen planus and that topical agents are unlikely to cause serious side effects. In this study, Table 2 shows that topical steroid therapy tends to be associated with oral candidiasis. However, we found that oral candidiasis was not associated with topical steroid therapy according to multivariate and propensity score-matched analyses (Tables 2 and 4). Thus, topical steroid therapy for radiation-induced oral mucositis may not be associated with oral candidiasis *per se*, and oral candidiasis may be associated with severe oral mucositis or immunocompetence of the host.

The main strength of our study is that patient recruitment was performed among the total number of patients treated with radiotherapy in the head and neck region. Thus, our study could avoid selection bias. The studied patients were most likely homogeneous, because head and neck cancer patients comprised 91.7% of the cohort (Table 1), and all patients received the same oral management during radiotherapy of the head and neck region.

However, our study also had some limitations. First, only angular stomatitis (one clinical form of oral candidiasis) was not treated with antifungal agents. Thus, our results may underestimate the incidence of oral candidiasis. We did not diagnose oral candidiasis with microbiological tests because positivity for *Candida* is not diagnostic of an infection. Second, data on minimum lymphocyte counts and oral mucositis grade were not always available before the onset of oral candidiasis. Thus, a causal relationship was lacking.

In conclusion, this study has shown that the incidence rate of oral candidiasis was 25.0% among patients who received radiotherapy for head and neck cancer with oral management, and that oral candidiasis during radiotherapy was associated with a low lymphocyte count and severe oral mucositis. These findings suggest that oral candidiasis is associated with host defenses and oral mucosal barrier to infection. Moreover, topical steroid therapy did not influence the incidence of oral candidiasis. The oral cavity should be kept as clean as possible during radiotherapy. In addition to this, the prevention or inhibition of severe oral mucositis may reduce the incidence of oral candidiasis.

## Author contributions

**Data curation:** Madoka Funahara.

**Formal analysis:** Yumiko Kawashita.

**Investigation:** Yumiko Kawashita, Masako Yoshimatu, Noriko Nakao, Sakiko Soutome.

**Project administration:** Masahiro Umeda.

**Supervision:** Toshiyuki Saito.

**Writing – original draft:** Yumiko Kawashita.

**Writing – review & editing:** Yumiko Kawashita, Masahiro Umeda.
